# p53 Signaling in Cancers

**DOI:** 10.3390/cancers11030332

**Published:** 2019-03-08

**Authors:** Natalia Issaeva

**Affiliations:** Department of Otolaryngology, Head and Neck Surgery, University of North Carolina at Chapel Hill, Chapel Hill, NC 27599, USA; natalia.isaeva@med.unc.edu

This special issue on p53 explores different aspects of the significance of p53 in normal cells and in cancer. Currently, everyone working in the area of cell biology knows about the tumor suppressor p53. Indeed, p53 is an extraordinary multifunctional protein, regulating cell cycle, differentiation, inflammation [1], immune response, metabolism [2], hormone-induced processes [3], transcription, DNA repair, epigenome [4], senescence and autophagy [5]. The localization of p53 at active replication forks and the p53-dependent effects on DNA elongation implies the involvement of p53 in the process of DNA replication [6]. Two different reviews by J. Kasteri et al. and V. Marcel et al. focus on how p53 also regulates protein translation and synthesis [7,8]. 

Being a powerful inductor of apoptosis, p53 protein is maintained at a low level in normal cells. In response to diverse stress conditions, the p53 level is stabilized through different posttranslational modifications, which often regulate p53 binding with its natural destructor hdm2 and create multiple feedback loops. Thus, the stress-responsive kinase p38 MAPK phosphorylates p53 at serine 33 and serine 46, which contributes to p53 stabilization and activation. On the contrary, the activated p53 induces Wip1 phosphatase expression, facilitating a negative regulatory feedback on p38 MAPK/p53 signaling [9]. 

p53 is a potent transcription factor and p53-dependent transcription is regulated by many cofactors. For example, Junctional Mediating and Regulating Y protein (JMY) together with p300 binds the phosphorylated p53 and enhances its transcription activity, leading to the selective induction of apoptosis [10]. Interestingly, in addition to the potent stimulation of transcription, the activated p53 can efficiently downregulate genes that are involved in telomere maintenance; DNA repair; centromere structure [11]; and telomere shortage. In turn, deficient DNA repair activates p53 that creates a positive feedback loop, which is tightly controlled in normal cells via hdm2-mediated p53 degradation. 

The p53 protein provides a substantial difference between normal and cancer cells. Wild type p53 functions are almost universally disabled in human cancer. The inactivation of the p53 occurs through varied mechanisms: directly by mutation, through binding to viral proteins or indirectly as a result of alterations in genes whose products either activate, regulate or carry signals from p53. For example, in the deadly malignant brain human tumor glioblastoma, the mutations in the *TP53* gene are detected in ~27% of tumors, while the most frequent genetic alteration in tumors carrying wild type p53 involves the deletions in the hdm2 negative regulator CDKN2A/ARF (57%) or *HDM2* gene amplifications (~11%) [12]. Recent studies established that in addition to protein-regulators, p53 is controlled by miRNAs in tumors [13]. The interaction between p53 and hdm2 has been intensively investigated, resulting in the development of hdm2 inhibitors. Nutlin family hdm2 antagonist idasanutlin is currently in clinical development for acute myeloid leukemia (AML). K. Seipel et al. present data showing that the combination of idasanutlin and MEK inhibitor cobimetinib is an effective treatment against AML with wild type p53 and elevated FLT3 and hdm2 levels [14].

Inactivating mutations in the *TP53* gene occur in around 50% of all human tumors and are associated with rapid tumor progression and resistance to anticancer therapy. Emerging data firmly support oncogenic roles for mutant p53 and together with stabilization of mutant p53 in tumors, the data suggest that targeting of mutant p53 may be a promising anticancer treatment strategy. The reviews by R. Schulz-Heddergott and U. Moll [15] and S. Yamamoto and T. Iwakuma [16] outline several pathways of mutant p53 regulation in cancer and discuss approaches that are aimed at targeting or reactivating mutant p53. C. Deben et al. used the most advanced p53-reactivating small molecule PRIMA-1 (APR-246) [17] to overcome hypoxia-induced cisplatin resistance in non-small cell lung cancer cells [18]. The other promising PRIMA-1 combinations with chemotherapeutic drugs and our understanding of how PRIMA-1 works in cells were evaluated in detail by A. Perdrix et al. [19]. S. Kogan and D. Carpizo discuss novel zinc-deficient mutant p53-reactivating function of zinc metallochaperones, thiosemicarbazones [20]. They established a two-step mechanism that first includes the restoration of the wildtype p53 protein structure by recreating zinc binding and second focuses on the activation of p53 through posttranslational modifications [21].

Recently, it has been shown that overexpressed mutant p53 protein can form aggregates in vitro and in vivo, contributing to its oncogenic function and cancer progression. M. Kanapathipillai discusses prospective therapeutic approaches targeting mutant p53 aggregation in cancer [22]. In addition to mutant p53, some p53 isoforms exert the “gain-of-function” effects although the molecular pathways that are affected by these isoforms are still not completely understood [23].

As it is typical for the majority of oncogenes, mutant p53 not only provides tumors with growth advantage, but also renders these tumors sensitive to certain stimuli. Thus, the usual dysregulation of the G1/S checkpoint in the mutant p53 cancer cells results in another cell cycle checkpoint, the G2/M, being exceptionally critical for the survival and growth of such tumor cells. The research article by X. Meng et al. [24] explores the use of the WEE1 inhibitor that abrogates the G2/M checkpoint by preventing the WEE1-mediated phosphorylation of cdc2 at tyrosine 15 in combination with olaparib or gemcitabine to efficiently target gynecological mutant p53 cancer cells.

The oncogenic activity of cancer-causing viruses largely depend on the viral ability to inactivate wild type p53. M. L. Tornesello et al. explain how the Epstein–Barr virus, the high-risk human papillomavirus and the hepatitis C virus target the function of wild type p53 [25]. R. Aloni-Grinstein et al. provide additional functional links between p53 and viruses, highlighting how viruses manipulate p53 signaling pathways to promote their life cycle [26].

In summary, the special p53 issue covers a substantial portion of the existing knowledge and latest accomplishments in this persistently important field.
